# Shy or bold, all get caught: Two active capture methods show no behavioural bias in a large herbivore

**DOI:** 10.1371/journal.pone.0351124

**Published:** 2026-06-24

**Authors:** Rudy Brogi, Francesca Brivio, Sabrina Manera, Bastien Saladini, Marco Apollonio

**Affiliations:** 1 Department of Veterinary Medicine, University of Sassari, Sassari, Italy; 2 National Biodiversity Future Center (NBFC), Palermo, Italy; 3 Research Institute of Wildlife Ecology, Department of Interdisciplinary Life Sciences, University of Veterinary Medicine of Vienna, Vienna, Austria; Instituto Federal de Educacao Ciencia e Tecnologia Goiano - Campus Urutai, BRAZIL

## Abstract

Behavioural studies often assume that sampled individuals represent the broader population, yet behaviour-dependent capture bias can distort behavioural and ecological inferences. While more risk-prone individuals are likely to be overrepresented in captured samples, the potential for bias in wild animals, particularly large mammals, remains unclear due to the inherent difficulties in assessing it. This study tested whether chemical immobilisation and drop nets yielded samples of Northern chamois (*Rupicapra rupicapra*) representative of the population with respect to ecologically relevant behaviours related to the individual exposure to risks. We recorded the spontaneously exhibited behaviours of 25 free-ranging adult chamois previously captured and tagged, alongside their non-captured counterparts. First, we assessed the repeatability of key behaviours with expected positive (eating, walking, aggression) or negative (standing, resting, group centrality) associations with risk exposure in captured chamois, to evaluate their individual consistency and suitability as indicators of inter-individual behavioural variation. Eating, standing, resting, and group centrality were significantly repeatable, while walking and aggression were not. Second, we compared the frequency of these repeatable behaviours across chamois being captured using either method and their non-captured counterparts observed in the same sessions, controlling for demographic and environmental confounding factors. Both frequentist likelihood ratio tests and Bayesian analyses provided strong evidence against the presence of capture bias, indicating that both methods yielded a behaviourally representative sample in terms of eating, standing, resting, and group centrality. With this first explicit assessment of capture bias in a large mammal, we challenge the assumption that capture systematically skews behavioural data in wild animals, demonstrating that chemical immobilisation and drop nets can provide unbiased samples of behavioural diversity in chamois, and possibly in other large herbivores. Given the importance of minimising sampling biases in ecological research, we highlight the good opportunity to validate capture methods before extrapolating behavioural and ecological inferences.

## Introduction

Behavioural and ecological studies typically assume that sampled individuals are representative of the broader population and the violation of this assumption would introduce sampling biases that can distort conclusions and even affect conservation efforts [1,2]. Since many research questions entail the need for capturing animals to gather otherwise inaccessible data on behaviour, genetics and physiology [3–6], one often-overlooked source of bias arises from consistent individual differences in behaviour, possibly influencing an individual’s likelihood of being captured. Such behaviour-dependent biases can systematically skew research findings not only about behaviour itself, but also about population structure and ecology, because behaviour is often correlated (functionally and/or genetically) with physiological and life-history traits. As a result, non-random sampling of behavioural phenotypes can bias demographic inferences, including population size estimates, and systematically shift estimates of trait means and distributions [1].

Previous work has mainly addressed this issue in the framework of animal personality and behavioural syndromes [7], testing whether certain personality traits are disproportionately represented in captured samples [8–10]. However, because capture bias is driven by behavioural outcomes that affect capture probability rather than by their ultimate causes, it follows that any behavioural pattern consistently expressed within individuals can generate bias if it alters exposure to trapping or other risks. Risk-prone and more active individuals, typically more likely to undergo capture procedures, can indeed become overrepresented in captured samples [2,9]. The extent of this bias typically depends on the capture method employed: passive methods, such as baited traps, rely on the animal’s voluntary entry and often oversample those individuals that are less fearful of artificial apparatuses [11,12]. Conversely, active methods, which involve direct human action to physically restrain or chemically immobilise an animal, are generally expected to depend less on the animal’s immediate behaviour at the moment of capture and may therefore provide a more representative sample of the population [13]. For instance, hand nets captured individuals exhibiting greater variance in boldness in three-spined sticklebacks (*Gasterosteus aculeatus*) compared to passive traps, which were biased towards bolder and more sociable individuals [13]. However, capture bias has been reported for both active and passive methods [9,10], whereas other work found no significant biases regardless of the method used [8]. This mixed evidence underscores the relevance of explicitly testing behavioural representativeness across capture techniques and contexts. Beyond methodological challenges, other intrinsic factors such as sex and age can influence the probability of being trapped, with males and juveniles often being more exploratory and thus more likely to be captured by passive trapping than females and adults, respectively [14–16].

While these studies provide valuable insights into capture bias, fully assessing its extent remains inherently challenging, as direct comparisons between captured and non-captured individuals would be necessary to accurately assess its effects. However, behavioural data often depend on capture itself, either to facilitate behavioural assessments under controlled conditions (e.g., [17,18]), mark and deploy tracking devices (e.g., [5,19,20]), or, rarely, both [21]. To circumvent this issue, most studies compare individuals captured using different methods [8,9,13,22] or analyse recapture probabilities in capture-recapture frameworks [10,16,23]. Yet, these approaches still rely exclusively on the trappable portion of the population. Direct comparisons between captured and non-captured individuals are rare and typically confined to controlled environments (and notably, still among pre-captured samples of individuals, e.g., [12,15]), leaving substantial gaps in our understanding of capture bias under natural conditions (but, for naturalised fish populations, see [1]).

Behavioural capture bias has been investigated across a broad taxonomic range with studies testing whether particular behavioural patterns are over-represented in captured samples of birds, reptiles, fish and small-to-medium-sized mammals [8,9,12–15]. In contrast, despite its potential importance for large-bodied species, capture bias has seldom been explicitly tested in ungulates and other large mammals (but see Reale et al., [10]), particularly under field conditions and across capture methods, likely owing to their wide home ranges, the difficulty of reliably recognising individuals, and the logistical challenges of assessing consistent behavioural differences in the wild [24]. Given the frequent use of capture techniques in large mammal studies for ecological research, evaluating potential sampling biases is crucial for ensuring robust ecological conclusions. Moreover, because such biases may affect the efficiency of capture-based interventions such as removals and translocations, their assessment is also fundamental for improving wildlife management and conservation strategies.

This study investigates whether two active capture methods, such as chemical immobilisation and vertical drop nets, used to capture Northern chamois (*Rupicapra rupicapra*), yield representative samples of the population with respect to the ecologically relevant behaviours. To test the hypothesis of capture procedures introducing behavioural biases in the sample of captured individuals, we compared behavioural patterns of 25 captured chamois with their non-captured conspecifics by means of direct observation of their spontaneous behaviours, focusing on those related with the exposure to risks, using intra-group comparisons to account for unrecognizable individuals and controlling for demographic and environmental confounding effects. First, to confirm that the selected behaviours were suitable for evaluating inter-individual behavioural variation, we tested whether the recorded behavioural patterns varied significantly and consistently among captured individuals. Second, we tested whether the behavioural patterns of the captured individuals significantly differed from those of their non-captured counterparts. If capture methods introduce bias, we predicted that collared individuals should exhibit distinct behavioural patterns compared to non-collared individuals. Beyond assessing the presence of capture bias in our case study, we aim to provide a practical methodological framework for evaluating the occurrence and potential consequences of capture-related sampling biases in field studies of wild animals. By combining non-invasive direct observations with within-group comparisons in free-ranging individuals, this approach allows researchers to assess whether capture procedures yield a representative sample of behavioural variation, thereby strengthening the validity of behavioural and ecological inferences used in research, conservation, and management.

## Methods

### Ethics statement

The study complied with all national and regional ethics and animal welfare regulations, and adhered to the ASAB/ABS Guidelines for the Ethical Treatment of Non-human Animals in Behavioural Research [25]. Capture and handling protocols were approved by the Italian Institute for Environmental Protection and Research (ISPRA, protocol no. 07930/2023) and the Veneto Regional Administration (decree no. 166–11/05/2023).

### Study area

**Fig 1:**
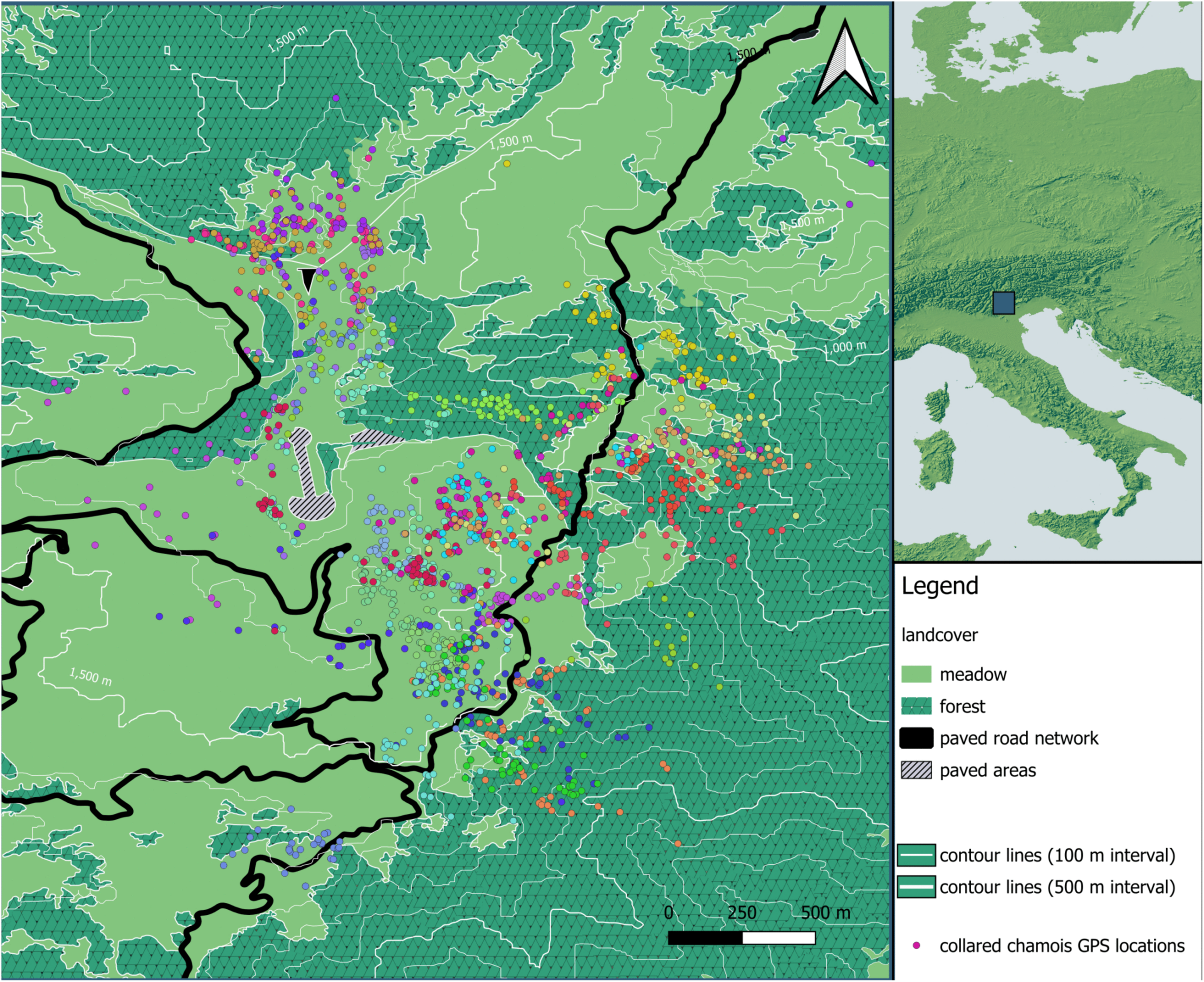
Study area on the Monte Grappa Massif, showing land cover and the GPS locations recorded during the behavioural observation period (May–June 2024) by collars deployed on the 27 captured Northern chamois. To improve readability, only locations recorded at 12:00 each day are shown. The inset shows the location of the Monte Grappa Massif in Europe. Land-cover data: Regione del Veneto, Banca Dati della Copertura del Suolo (Italian Open Data Licence, IODL 2.0). Hillshade (inset): European Environment Agency (EEA), Hillshade Europe DEM (CC BY 4.0).

This study was conducted in the Monte Grappa Massif (Venetian Prealps, North-East Italy; 45.873103N, 11.808166E, Fig 1), a 400 km² mountainous complex reaching a maximum elevation of 1 775 m a.s.l.. The massif is geographically distinct, bordered by the Piave and Brenta rivers and spanning the provinces of Treviso, Belluno, and Vicenza. The environment transitions from mixed deciduous forests at lower elevations (600–1 300 m) to a mosaic of beech-conifer forests and open areas between 1 300 and 1 650 m. Above 1 650 m, the landscape is dominated by alpine meadows and rocky outcrops (Fig 1). The climate is suboceanic pre-Alpine, with snowy winters, mild summers, and frequent fog formation on southern slopes. Human presence in the area is relatively high during spring and summer due to mountain sports such as hiking, climbing, biking, and paragliding, but is not negligible even throughout autumn and winter. This is facilitated by a relatively well-developed paved road network, which allows year-round access to the mountain top by car. Meanwhile, permanent human settlements are virtually absent.

After their extinction in historical times, Northern chamois were reintroduced to the area between 1995 and 2006 using wild individuals translocated from other regions of the Italian Alps (Maritime and Dolomite Alps). The historical extirpation of chamois from the massif is plausibly linked to overexploitation during the period when the World War I front crossed this area. Chamois primarily inhabit higher elevations above 1 300 m. Since 2016, they face predation from grey wolf (*Canis lupus*), following the species’ natural recolonization of the Alpine region after historical declines caused mainly by persecution linked to livestock conflicts.

### Capture of chamois

We captured 27 adult chamois during five three-day capture sessions conducted between June 2023 and March 2024 as part of a research project on chamois spatial ecology that aimed to obtain a broadly balanced sex ratio in the sample. We used two distinct active methods, applied in separate sessions: chemical immobilisation during three sessions starting on 20 June, 18 July and 3 December 2023, and vertical drop nets during two sessions starting on 26 September 2023 and 25 March 2024.

Chemical immobilisation was carried out by trained ground shooters of the local Provincial Police Corps, using darting air rifles. A veterinarian prepared darts, containing a combination of approximately 25 mg of ketamine, 1.25 mg of medetomidine, and 1.00 mg of acepromazine, corresponding to a dosage of 1 mg/kg ketamine, 0.05 mg/kg medetomidine, and 0.04 mg/kg of acepromazine for an estimated 25 kg chamois. Capture sessions were concentrated around sunrise and sunset, when chamois were most detectable. These periods were defined operationally as approximately 1 h before to 2 h after sunrise and 2 h before to 1 h after sunset, respectively, with each session lasting ~3–4 h. Capture efforts involved three to five shooters systematically patrolling open meadows within the study area, aiming to approach chamois within 30–40 m before firing at the thigh. Meanwhile, 10–15 observers, positioned at vantage points and equipped with binoculars, monitored chamois movements and assisted in directing shooters via radio communication. Once darted, each chamois was closely observed until the anaesthetic took effect (~3–7 minutes), after which a team of two experienced handlers and one veterinarian approached and blindfolded the immobilised animal. The veterinarian continuously monitored heart rate, oxygen saturation, respiratory rate, and rectal temperature during all the subsequent phases of manipulation in order to monitor the health conditions of the captured animals. The operators recorded standard biometric measurements (i.e., hind foot length, shoulder height, and total body length), weighted the chamois with a digital scale, and estimated age based on horn growth rings to ensure that the subject was adult (older than 2 years). After that, the operators fitted the chamois with a GPS collar (Vectronic Aerospace Vertex Fusion or Vertex Plus, 0.60–0.75 kg depending on model and belt length) and tagged it with one or two permanent plastic ear-tags. Unique colour combinations of collars and ear-tags allowed for subsequent individual identification from a distance. At the end of the manipulation procedure, the veterinarian immediately administered the antagonist atipamezole hydrochloride (0.25 mg/kg) to reverse medetomidine and accelerate recovery. Handling time ranged from 15 to 25 minutes. Using chemical immobilisation we captured, collared, and ear-tagged a total of 11 adult males, as this method was deliberately applied only to males. This choice was constrained by females’ longer flight initiation distance, which often prevented operators from approaching within the darting range (30–40 m), and by the limited period in which females could safely receive anaesthetic drugs, as pregnancy and lactation restricted the feasible use of this technique to just a few months per year, during which their tendency to remain near steep rocky areas would still have made sedation unsafe.

Vertical drop nets were deployed along the forest edge, targeting flight routes that had been identified beforehand from repeated observations of chamois groups fleeing from the same meadows and consistently using the same escape corridors (S1 File). Accordingly, net-line locations and operator positions were pre-selected in advance (including approach paths that kept operators concealed), but nets were only installed once one or more groups had been spotted in an open meadow, following the same capture schedule described for chemical immobilization (sessions conducted over three consecutive days, concentrated around sunrise and sunset). Nets were 2.20 m high and placed in lines ranging from 150 to 300 m in length, depending on terrain conditions, and were typically set up by four–eight operators when chamois were at least ~300 m from the forest. Once the nets were in place, a team of five to seven operators conducted a controlled drive, slowly advancing towards the group to encourage their movement towards the net line. Net captures could involve single individuals or multiple animals simultaneously, including entire groups (typically up to five–six adults, with occasional kids). Upon entanglement, chamois were immediately manually restrained, blindfolded and tied with soft ropes by experienced operators to impede injuries and limit stress, before undergoing the same biometric measurements, collaring and tagging procedures as chemically immobilised individuals, but with no drugs being administered. Since these net-captured chamois were not anaesthetised, we shortened handling times to 10–15 minutes to minimise handling stress. During drop-net operations, groups containing females were prioritised when encountered on suitable meadows, although males were also captured when present in the targeted groups. When multiple individuals were captured simultaneously in the same net, all captured adults were processed and marked during the same capture event. Using drop nets, we captured, collared, and ear-tagged a total of 16 adult chamois (12 females and 4 males), along with an incidental bycatch of 1 yearling and 5 kids, which were not fitted with collars and immediately released. No injuries were observed during capture or handling.

### Behavioural observations

Behavioural observations were conducted in May and June 2024, allowing at least two months for chamois to recover from capture and ensuring that recorded behavioural patterns were not influenced by capture-related stress [5,19]. Observations were restricted to a relatively homogeneous 5 km² area at the top of the massif, where all collared chamois were residing. Data collection was conducted exclusively in open meadows and during daylight hours, with most observations occurring from 11.00 AM to 08.00 PM, when weather conditions, particularly the absence of fog, facilitated data collection.

Observations were conducted on groups containing at least one collared and one non-collared adult chamois. Collared individuals (hereafter “focal”) were initially listed in capture order, with selection following a rotating priority system to prevent operator bias. Two operators used GPS data and, occasionally, a Very High Frequency (VHF) beacon to locate focal individuals. Once identified by their unique collar and ear-tag colour combination, observations commenced if the focal was accompanied by at least one uncollared adult of the same sex in the same group (as defined by the 50 m chain rule, where each chamois in the group is within 50 m of any other chamois). The location and start time of each observation session were recorded. The coordinates of the observation were later used to quantify the proximity to the nearest hiking trail, as the Euclidean distance (m) between each observation location and the closest segment of the official trail network provided by the Veneto Region (https://idt2.regione.veneto.it/idt/webgis/viewer?webgisId=216). Groups were observed from a distance of at least 100 m using binoculars for a minimum of 30 minutes. If another collared individual was in sight at the end of this period, it became the new focal, and a new observation session began. Otherwise, the initial focal was observed for an additional 30 minutes before concluding, for a maximum observation duration of 60 minutes per focal individual. After an observation session ended, observers could either initiate a new session on another collared individual or terminate observations.

Behavioural data were collected via instantaneous scan sampling at 3-minute intervals, recording mutually exclusive behavioural states and intra-group spatial positioning (see Table 1 for a detailed description) of all adult chamois of the group. Relative intra-group spatial positioning was recorded live and visually estimated using a virtual grid, then classified into a four-level group centrality index ranging from 1 (peripheral) to 4 (central; see S2 File for details). The collared status (yes or no) of each individual, its sex, and its identity (for collared chamois only) was also noted. Of the seven recorded behaviours, only relative intra-group positioning, rest, eat, stand, walk, and non-confrontational aggressive behaviour were analysed, as confrontational aggressive behaviours were too rare to model (<20 occurrences), resulting in insufficient data for reliable parameter estimation and inference. To ensure coding reliability, 70% of the behavioural records from the primary observer were compared against a continuous live coding session performed on the focal individual by a second observer using the BORIS v. 8.25.4 mobile application. Cohen’s kappa values summarising inter-observer agreement are reported in Table 1, with values of 0.40 to 0.60, 0.60 to 0.75, and > 0.75 representing fair, good, and excellent agreement, respectively [26].

**Table 1:** Description of the analysed behaviours in Northern chamois, inter-observer agreement indices, relationship with the exposure to risks, and supporting references.

behaviour	description	Cohen’s kappa	relationship with the exposure to risks	supporting references
rest	the subject lies on its chest and abdomen, or on its side. legs may be underneath the body or stretched out	0.97	negative, as resting individuals are less exposed to risks and may detect them more efficiently	[27,28]
eat	the subject grazes with its head mostly down	0.78	positive, as the head-down position during foraging increases exposure to predation risk in grazers	[29–31]
stand	the subject remains with its head held above the horizontal line of the back	0.55	negative, as grazers adopt a vigilant head-up posture to mitigate predation risk	[29–31]
walk	the subject moves at any speed less than galloping	0.36	positive, as greater spatial movements are associated with increased detectability and risk	[28,32,33]
non-confrontational aggressive behaviour	the subject marks, rubbing its head or horns on a plant to deposit scent	0.85	positive, as engaging in intra-specific aggressive behaviours may reduce vigilance toward predators and other risks	[34,35]
confrontational aggressive behaviour	the subject displays at least one of the following behaviours to another chamois: stare, rush, chase, head down, hook, neck up	*rare*	positive, as engaging in intra-specific aggressive behaviours may reduce vigilance toward predators and other risks	[34,35]
intra-group centrality	spatial position of the subject within the group, visually estimated on a virtual grid	/	negative, as occupying peripheral positions entails higher predation risk but potentially greater foraging opportunities	[36,37]

A total of 69 independent observation sessions were conducted on 25 collared chamois (11 females, 14 males) across 22 observation days, as one female and one male were never located in open areas. As multiple collared individuals were occasionally observed within the same group and could be monitored simultaneously, this amounted to 135 individual observation sessions. Each collared individual was observed in an average of 5.40 ± 2.89 independent sessions, accumulating a mean total of 3.68 ± 1.91 hours per chamois (mean ± SD). The observed groups comprised an average of 3.84 ± 2.20 adult chamois per group, with collared individuals accounting for a mean proportion of 0.45 ± 0.20 of the total group size.

### Statistical analyses

#### Repeatability of collared chamois’ behaviours.

All statistical analyses were conducted using R version 4.4.2 [38].

In this first step, we aimed to determine which behaviours varied significantly and consistently across individuals, indicating their suitability as indicators of inter-individual behavioural variation. Since this assessment required knowledge of individual identity, the dataset for this analysis included only behavioural records of collared (and, thus, captured) chamois. These records were compiled by merging all observations of collared individuals at a 3-minute resolution. The dataset included 1 346 records, separated among 69 different observation sessions on a total of 25 collared chamois. The dataset included the group centrality index (either 1, 2, 3 or 4) as a four-level ordered variable, whereas eat, rest, stand, walk and non-confrontational aggressive behaviour were coded as binary variables, informing whether the individual was, or was not, exhibiting the specific behaviour.

We identified the behaviours that were expressed in a repeatable manner within individuals (similar frequency over observation sessions) by calculating repeatability, i.e., a variance-based measure that quantifies the proportion of phenotypic variation attributable to among-individual differences [39]. Repeatability was estimated separately for each behaviour by comparing within-individual variation in its likelihood to be exhibited to overall among-individual variation, using the rptR package in R [40]. A binary distribution was used for binary behaviours, while a Gaussian distribution was applied for the group centrality index. Adjusted repeatabilities were calculated by including observation session id as an additional random effect (alongside individual identity) in the rpt() formula, as well as fixed control effects that could influence chamois behaviour, specifically: sex, group size, time from noon, and proximity to the nearest hiking trail. Time from noon was treated as a continuous variable, the absolute difference in hours from noon, to better capture potential reductions in activity during the central part of the day. This approach controlled for potential influences of inter-sexual differences, group size, circadian activity rhythms, and human disturbance, respectively, on chamois behaviour [41–43]. P-values < 0.05 indicated significant repeatability of the given behaviour, suggesting that certain individuals consistently foraged, stood, rested, walked, and/or displayed non-confrontational aggressive behaviours more or less frequently than others, while controlling for sex, group size, time of day, and local disturbance.

Only behaviours exhibiting significant within-individual repeatability (i.e., eat, stand, rest, and group centrality index) were included in the subsequent analysis. For these behaviours, individual scores were obtained as the individual random-intercept estimates from full models analogous to those described above, fitted as a linear mixed model (LMM) for the group centrality index and as generalized linear mixed models (GLMMs; binomial error distribution with logit link) for the binary behaviours [44]. Models were fitted in R using the lmer() and glmer() functions of the lme4 package. Finally, to test whether individuals showing higher risk exposure in one behavioural dimension also tended to do so in others, pairwise associations among these individual scores were evaluated using Pearson correlation coefficients.

#### Capture bias testing.

In the second step of the analyses, we aimed to assess whether behavioural patterns differed among chamois captured via chemical immobilisation, those captured using drop nets and those that had not been captured at all. While our primary focus was on comparing captured versus non-captured individuals, this approach allowed us to also evaluate potential differences between the two capture methods. We thus used the complete set of behavioural records, including those referred to the non-collared individuals. Since the latter could not be individually recognised, we addressed this limitation by adding the observation session id as a random effect in the model. This allowed us to compare the behaviour of the chamois belonging to the three groups during the same observation session and within the same environmental and temporal context, effectively accounting for the nested structure of the data.

The dataset included 3 186 behavioural records, collected during the 69 observations, with rest, stand, and eat included as binary variables and the group centrality index as a four-level factor, as described above. Prior to fitting the models, we screened all predictors for collinearity (Pearson coefficient |rp| > 0.6) and multicollinearity (Variance Inflation Factor, VIF ≥ 3, [45]), but no issues arose. After this, we z-transformed the group size, the time from noon and the proximity to the nearest hiking trail to a mean of zero and a standard deviation of one to get comparable estimates and easier interpretable model results [46]. We added a further variable named “capture”, a three-level factor to accounting for possible differences between chamois being captured by means of chemical immobilisation, vertical drop nets, or not being captured at all. Separately for each binary behaviour, we ran a GLMM with a binomial distribution, fitted by means of the glmer() function of the lme4 package [44], with the likelihood of exhibiting the behaviour being considered as response, sex, group size, time from noon, and proximity to the nearest trail as control fixed effects, “capture” as main predictor, and the observation id as random intercept. In order to consider the possible differences of sensitivity to the human disturbance across individuals being captured with different methods or not being captured at all, we also included the interaction between “capture” and the proximity to the nearest trail. To keep type I error rate at the nominal level of 0.05, we included the random slope of “capture” within observation id [47,48].

As an overall test on the significance of “capture” on the exhibited behaviours and to avoid ’cryptic multiple testing’ [49] we compared the fit of the full model as described above with that of a null model lacking the “capture” main predictor and its interaction with the trail proximity but being otherwise identical, by means of a likelihood ratio test. We repeated the whole procedure for the group centrality, using an LMM, implemented with lmer() function, instead of a binomial GLMM.

Finally, since we were interested in testing the absence of behavioural differences across individuals being captured with the different method or not having been captured at all, we employed a Bayesian approach because it allows for the direct quantification of evidence in favour of the null hypothesis [50,51]. This approach enabled us to test the absence of an effect rather than relying solely on rejecting or failing to reject an alternative hypothesis. We used the same dataset described above to test whether the likelihood of exhibiting each considered behaviour differed across the three levels of “capture” using a Bayesian framework (BayesFactors R package, [52]). The analysis accounted for the same control predictors described above as fixed effects, with random variability associated with observation session id and its interaction with “capture” (i.e., random slope of capture within observation sessions) included as random effects. Analogously to what described in the linear model analyses, a full model, which included capture, was compared against a null model that excluded it but was otherwise identical. Model comparisons were performed by calculating Bayes Factors (BF), which quantify the evidence for including capture as a predictor. Bayes factors were computed using the package’s default Jeffreys–Zellner–Siow (JZS) prior specification for linear models (BFlinearModel, JZS). A BF greater than 1 supports the hypothesis that capture activities are biased toward specific behavioural patterns, while a BF less than 1 supports the null hypothesis that the considered behaviour does not differ across chamois that were captured using chemical immobilisation, drop nets, or not captured at all [53,54]. For interpretation, we followed conventional guidelines for Bayes factors, treating BF < 1/3 as evidence in favour of the null and BF < 1/10 as strong evidence in favour of the null; values closer to 1 were considered inconclusive. The whole framework was repeated separately on rest, stand, eat, and group centrality score.

## Results

### Repeatability of collared chamois’ behaviours

We detected a significant repeatability for eat, stand, rest, and group centrality index. The repeatability significantly differed from 0 for eat (R = 0.10 ± 0.03, mean ± SE, *P* < 0.001), stand (R = 0.05 ± 0.02, *P* < 0.001), rest (R = 0.15 ± 0.04, *P* < 0.001), and group centrality index (R = 0.15 ± 0.05, *P* < 0.001). The models’ summaries also revealed significant effects of the control predictors, with a decrease of eat (β_time from noon_ = 0.69, *P* = 0.02) and an increase of rest (β_time from noon_ = −1.04, *P* = 0.008) in the central hours of the day, where ‘time from noon’ is the absolute number of hours from 12:00. Moreover, a significant difference between sexes emerged from the group centrality index model, with males occupying more peripheral positions compared to females (β_male_ = −0.66, *P* = 0.006). Conversely, the repeatability tests for walk (df = 1, χ^2^ < 0.001, *P* = 1) and non-confrontational aggressive behaviour (df = 1, χ^2^ < 0.001, *P* = 0.50) did not reveal significant individual repeatability.

**Fig 2:**
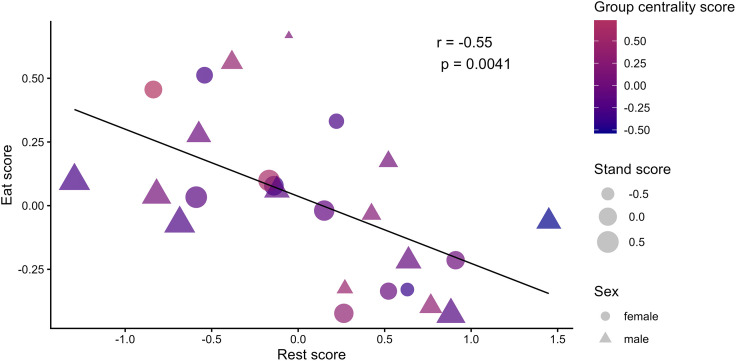
Individual behavioural scores for 25 Northern chamois captured, equipped with collars, and observed in Northeast Italy in 2023-2024. The x-axis represents the rest score, while the y-axis represents the eat score. Dot size corresponds to the stand score, dot colour represents the group centrality score, and dot shape indicates sexes. Individual scores for rest, eat, stand, and group centrality were obtained by means individual random intercepts of Mixed Models (see section Repeatability of collared chamois’ behaviours of the main text for more details).

The Pearson correlation analysis revealed significant negative relationships between rest and eat individual scores (*r*_rest-eat_ = −0.55, *N* = 25, *P* = 0.004), indicating that individuals resting more frequently tended to be less likely to eat. No significant correlations were found among the other individual scores. The overall distribution of the 25 individual chamois’ behavioural scores is represented in Fig 2.

### Capture bias testing

**Fig 3:**
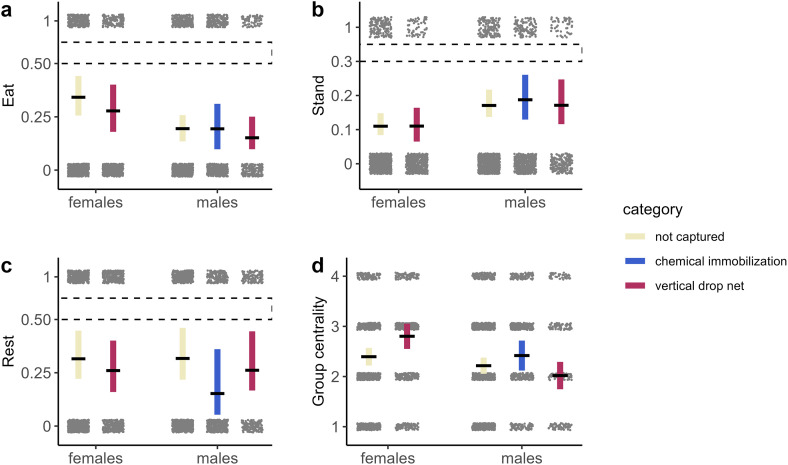
Variability in the behaviours of chamois (a) eating, (b) standing, (c) resting, and (d) group centrality, as predicted by the full models described in section *Capture bias testing* of the main text. Predictions are shown across the chamois captured using chemical immobilisation, using drop nets, and non-captured, separately for males and females. Vertical bars represent 95% confidence intervals (CI) obtained through bootstrapping, while grey dots represent the observed scan-sampled raw data. Rectangular areas enclosed by dashed lines indicate a break in the y-axis.

The likelihood ratio tests revealed no significant differences among the chamois being captured with chemical immobilisation, drop nets, and not captured at all, on any of the considered behaviours, including eat (χ² = 2.54, df = 4, *P* = 0.64), stand (χ² = 0.63, df = 4, *P* = 0.96), rest (χ² = 6.19, df = 4, *P* = 0.19), and group centrality (χ² = 4.69, df = 4, *P* = 0.32) (Fig 3).

Similarly, the Bayesian analyses provided an overwhelming evidence against a difference among individuals captured with different methods, or not captured at all, in the likelihood of exhibiting these behaviours, with BFs strongly supporting the null hypothesis for eat (BF = 0.0017 ± 2.84%), for stand (BF = 0.0009 ± 3.95%), for rest (BF = 0.006 ± 19.10%) and for group centrality (BF = 0.01 ± 27.11%).

## Discussion

Our results revealed significant and repeatable individual variability in four of the investigated behaviours within the study population, while also demonstrating that the two evaluated capture methods provided a reliable representation of this existing behavioural diversity.

The significant repeatability of eating, resting, standing, and group centrality index indicates that these behaviours were consistently expressed within individuals and significantly varied among them [39], supporting their suitability as indicators of consistent inter-individual behavioural variation in the population. In contrast, walking and non-confrontational aggressive behaviours did not exhibit significant within-individual repeatability, likely due to their relatively low frequency, at least when recorded through scan sampling. The negative correlation between resting and eating, along with their significant variation throughout the day (consistent with a summer bimodal activity rhythm, [41]), suggests their association with activity levels, although their relevance in characterizing individual differences in the activity behavioural trait would have required an independent confirmation. By contrast, we found no evidence of individual-level covariation between standing and group centrality (Table 1). This pattern suggests that these behaviours may not reflect a single underlying behavioural trait or syndrome but could instead represent individually repeatable trade-offs between different forms of risk-taking, as observed in other wild ungulates [24].

Regardless of their relationships with underlying behavioural traits, overall activity (time spent eating versus resting), standing behaviour, and group centrality remain key components of individual risk–resource trade-offs. More active individuals acquire more resources at the cost of increased energetic expenditure and exposure to risks [32,33,55]; standing entails a trade-off between foraging efficiency and vigilance [29–31]; and centrality provides safety at the cost of increased intraspecific competition [36,37]. Given their ecological relevance and consistent within-individual repeatability, investigating whether capture methods selectively sample individuals differing in these behaviours remains crucial to understanding potential biases in behavioural ecology studies on individuals and populations.

Contrary to most studies investigating capture bias in other systems [2,9,11,12], our comparison of captured and non-captured chamois, while accounting for sex differences, demonstrated the absence of such bias, in accordance with Michelangeli et al. [8], who used active capture methods on delicate skink (*Lampropholis delicata*) despite not explicitly comparing captured and non-captured individuals. Similarly, our findings indicating the absence of sampling bias may be attributed to the use of capture methods that are entirely active, meaning they do not require animals to engage in exploratory behaviour toward artificial objects, such as traps or other capture devices [8]. Nonetheless, our capture procedures still involved behavioural responses to the approach of human operators, which could have introduced biases based on individual differences in risk perception [2,9]. For drop nets, capture success was primarily driven by chamois flight responses, leading them to run towards the nets, regardless their tolerance to human operators, making the lack of a behavioural trait-skewed sampling unsurprising. Conversely, chemical immobilisation required operators to approach individuals at relatively short distances, raising the possibility of bias based on inter-individual variation in flight initiation distance. In this context, the longer flight distances of females compared with males (e.g., 46 and 108 m in males and females, respectively [56]) contributed to making them unsuitable targets for this method, which we deliberately applied only to males. This is consistent with the stronger risk avoidance generally shown by female chamois, in line with their sex-specific life-history strategy [57], and with our finding that females occupied safer, inner positions in mixed groups. However, female behaviour and flight distance were only part of the constraints preventing chemical immobilisation in females in this study: pregnancy and lactation limited the period in which anaesthetic drugs could be safely administered, while during the remaining suitable months females tended to remain near steep rocky areas, where sedation would have been unsafe. Therefore, although behavioural differences may have contributed to the impracticability of chemically immobilising females, our study system did not allow us to directly evaluate this potential source of sex-skewed samples.

However, our results showed that, within males, chemical immobilisation did not introduce any sampling bias, despite the 30–40 m darting distance being similar to or shorter than the male flight distances reported in other studies (46 m in Hamr [56], 103 m in Gander & Ingold [58]). A likely explanation is the relatively high and widespread tolerance to human proximity observed in our study population, likely facilitated by frequent exposure to recreational activities. Such exposure is known to habituate chamois to non-threatening human presence and has been shown to substantially reduce flight distances to approaching humans [59]. Accordingly, caution is warranted when extrapolating these results to other species, different ecological contexts, or study areas where chamois exhibit greater wariness toward human presence. Nonetheless, by explicitly testing behavioural representativeness across two active capture methods and by directly comparing captured with non-captured individuals in a free-ranging large mammal, our results suggest that while capture bias remains a concern that warrants consideration in large mammal ecological studies, its occurrence is not unavoidable, even when using methods that rely on individual tolerance to a human operator.

Our study entails some limitations. First, behavioural assessments were based on passive observations of spontaneously exhibited behaviours in natural conditions rather than controlled experimental setups. Since this approach may limit cross-study comparability and make relationships between observed behaviours and underlying individual traits uncertain [60], we acknowledge that our results cannot exclude possible biases in behavioural syndromes or traits. On the other hand, since the observation of spontaneous behaviours in non-manipulated contexts provides a more ecologically valid representation of behavioural variation within the population [61], our approach was well-suited for assessing potential capture bias in behavioural ecology patterns, particularly those related to the individual exposure to risks. Furthermore, although experimental approaches such as flight initiation distance trials could offer an alternative method to detect capture bias in large mammals, we deliberately avoided setups that might artificially alter chamois spatial movements, which could in turn bias the behavioural ecology analyses planned within the project (as shown by reduced use of open areas after flight distance experiments, [58]). Additionally, because we evaluated only two active capture methods, we could not directly compare active versus passive sampling approaches; such comparisons would be valuable to more broadly validate behavioural sampling bias within populations. Moreover, because chemical immobilisation was not applied to females, we were unable to assess potential capture bias for this method in them. Consequently, our conclusion that chemical immobilisation does not introduce behavioural bias applies only to males. Finally, our study focused exclusively on adult individuals of a relatively human tolerant population and was restricted to a specific period of the year. While this may limit the generalisability of our findings across life stages, ecological contexts and seasons, it also helped minimise potential confounding effects, allowing for a clearer interpretation of individual behavioural variation and its implications for capture bias.

This study provides a rare explicit assessment of capture bias in a wild, free-ranging large mammal, and, to our knowledge, the first to quantify it by directly comparing captured and non-captured individuals while also evaluating multiple active capture methods. We show that the two methods used for Northern chamois yielded a sample that accurately reflected the population’s behavioural diversity, particularly in behaviours linked to risk exposure and resource-acquisition trade-offs. However, to ensure robust conclusions about individual and population-level behavioural and ecological patterns, potential capture biases should be more systematically considered in behavioural ecology studies. Given its non-invasiveness for animals and practical applicability for researchers, we propose our protocol as a robust and transferable framework for behavioural studies involving the capture of wild animals, at least for species and environmental contexts where it is feasible to repeatedly observe the spontaneous behaviour of both captured individuals and their non-captured conspecifics.

## Supporting information

S1 File Schematic representation of vertical drop net capture procedures. (DOCX)

S2 File Description of relative intra-group spatial positioning assessment. (DOCX)
