# Supplementary material for: Shy or bold, all get caught: Two active capture methods show no behavioural bias in a large herbivore
Source: PLoS One. 2026 Jun 24;21(6):e0351124. doi: 10.1371/journal.pone.0351124 (PMC13293383; doi:10.1371/journal.pone.0351124)
Supplement: S1 File — (DOCX) [file pone.0351124.s001.docx]

**Supplementary Information S1**

**
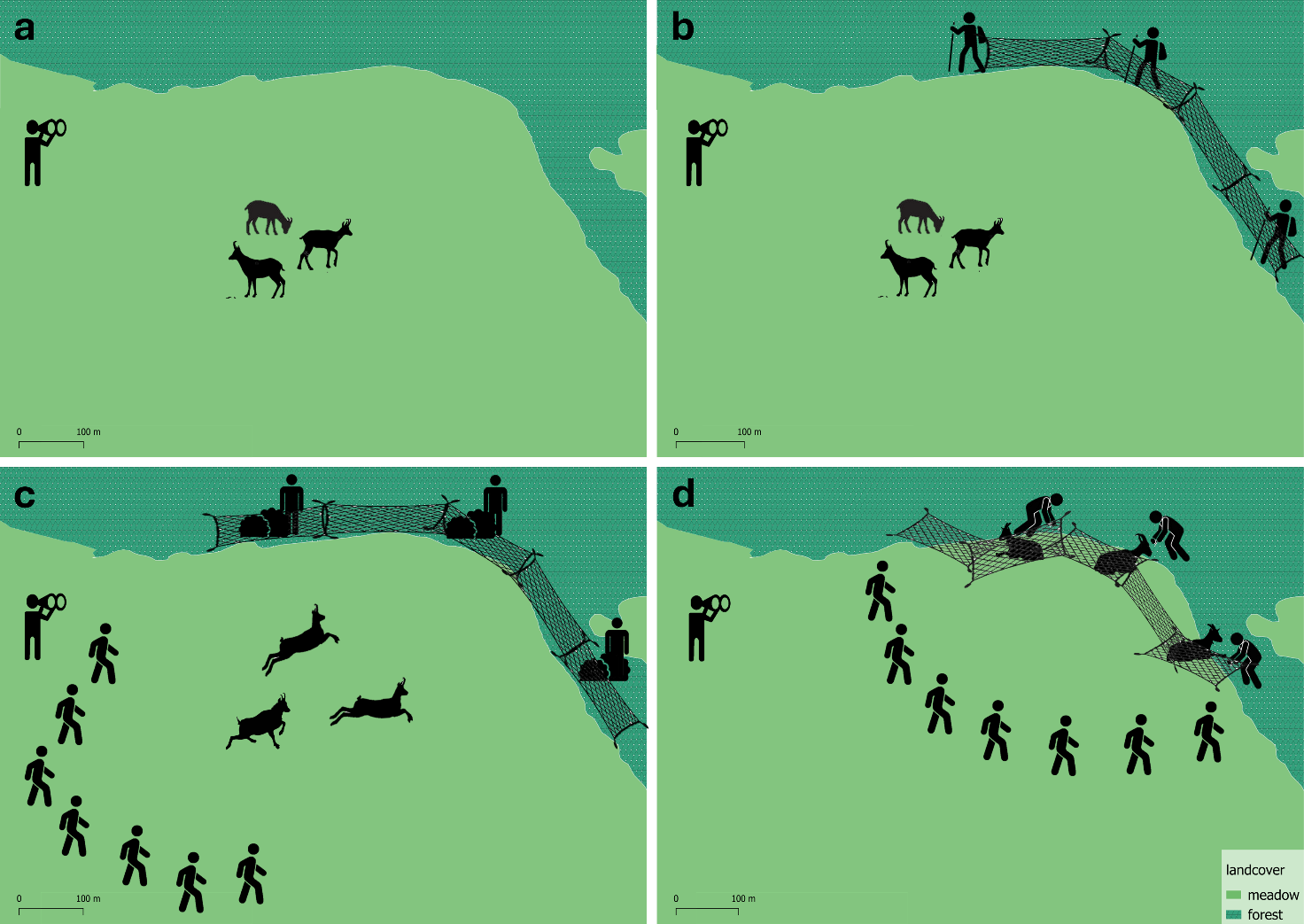
**

**Figure S1.** Schematic sequence of events during capture using vertical drop nets. (a) One chamois group is detected while foraging in an open meadow by an observer positioned at a distance. (b) After the group is sighted, operators deploy the vertical drop nets along a previously identified escape corridor at the forest edge, approaching and working from within the forest to remain concealed; observers coordinate timing and confirm that animals remain undisturbed. (c) A controlled, low-intensity drive is then initiated to direct the group towards the net line while the net-deployment team remains hidden near the nets. (d) Chamois become entangled in the nets and are promptly physically restrained by operators to minimise stress and handling time. Throughout all phases, observers and operators maintain continuous radio communication to synchronise actions and monitor animal movements.
