# Supplementary material for: Shy or bold, all get caught: Two active capture methods show no behavioural bias in a large herbivore
Source: PLoS One. 2026 Jun 24;21(6):e0351124. doi: 10.1371/journal.pone.0351124 (PMC13293383; doi:10.1371/journal.pone.0351124)
Supplement: S2 File — (DOCX) [file pone.0351124.s002.docx]

**Supplementary Information S2**


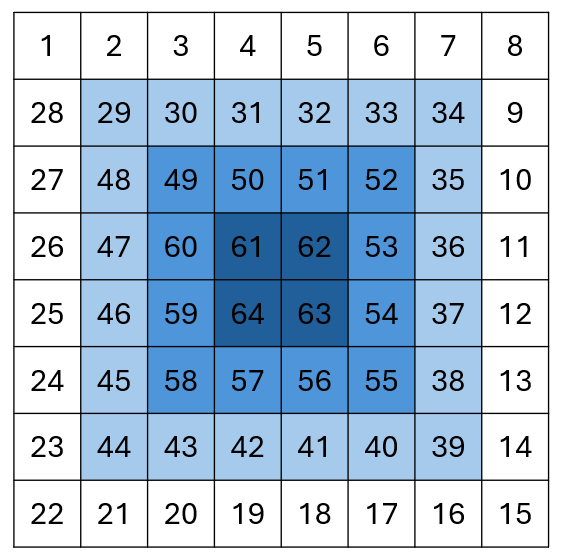
In this study, we employed the grid shown in Figure S2 to identify and record the spatial positions of individual chamois relative to all others in the group, including both collared and non-collared chamois. At each behavioural scan, the operator identified the group centroid as the visual balance point where individuals appeared evenly distributed around it (approximately corresponding to the estimated arithmetic mean of their spatial positions). The operator then virtually centred the grid on the group centroid and adjusted its dimensions so that the outermost individual in the group was positioned at the grid's outer edge (Figure S3), ensuring a standardised spatial scaling.

This approach ensured consistency in relative spatial assignments across different group sizes and structures, allowing comparability across measures in relative terms, without requiring absolute estimates of spatial distances. Since we aimed to use this relative group centrality as a proxy for exposure to risks (where central positions provide greater safety but limit foraging opportunities, see Table 1 of the main text for more details), we were not interested in absolute distances. Exceptions were made for groups in which all members were within 10 m of each other (a rare occurrence, observed only in small groups of 2 or 3 individuals). In these cases, to avoid introducing artificial variability in spatial categorisation that might not reflect meaningful differences in positioning, all individuals were assigned to the central grid cells.

Once the grid was positioned, the operator assigned each chamois to a grid cell reflecting its instantaneous spatial position relative to the group’s centroid. The grid was re-positioned at each scan (i.e., every 3 minutes).

**Figure S2.** Virtual grid used for estimating chamois’ relative intra-group spatial position.

Subsequently, the grid cell data were classified into a four-level group centrality index:

- 1 (peripheral, grid cell IDs 1-28)
- 2 (medium-peripheral, grid cell IDs 29-48)
- 3 (medium-central, grid cell IDs 49-60)
- 4 (central, grid cell IDs 61-64)


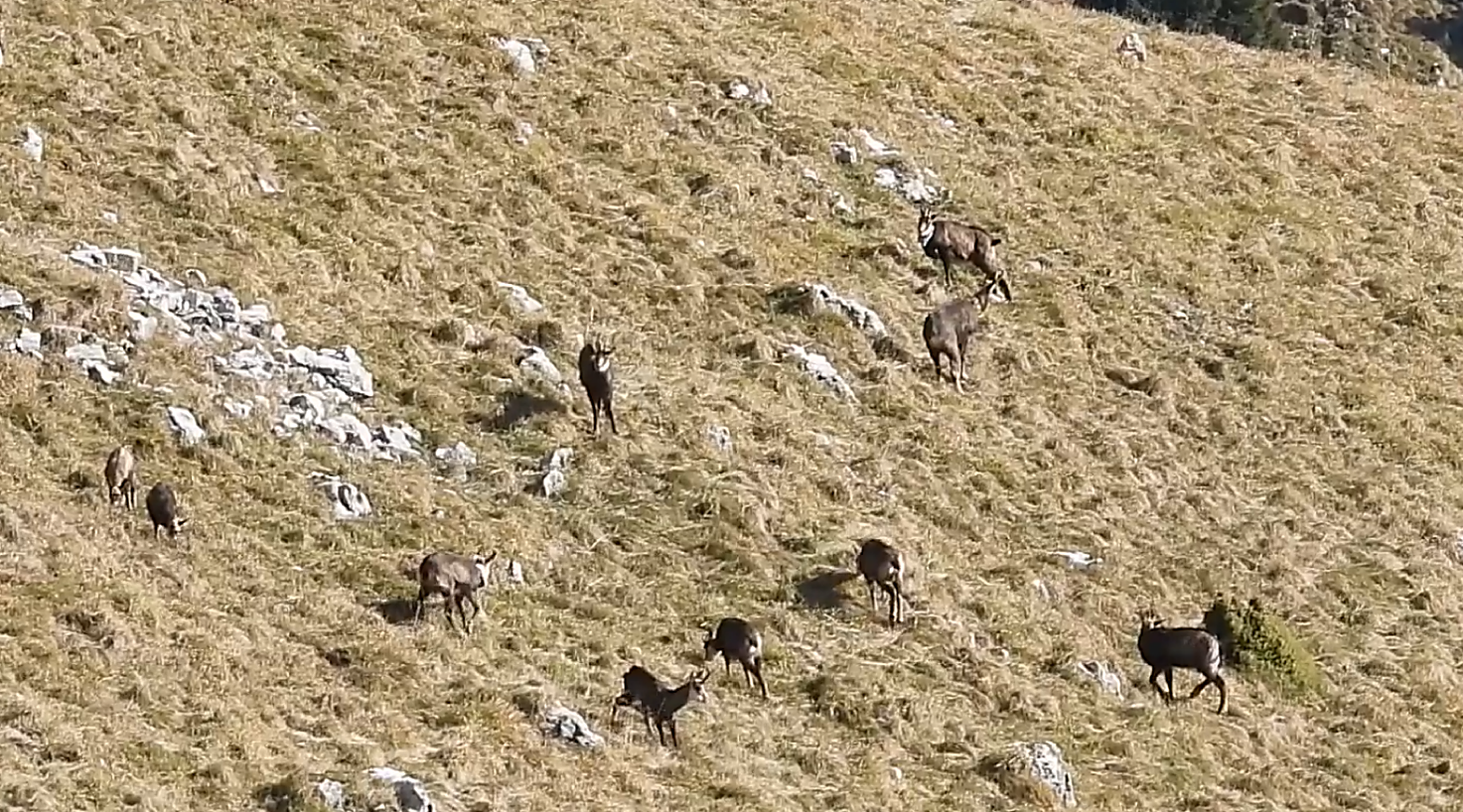

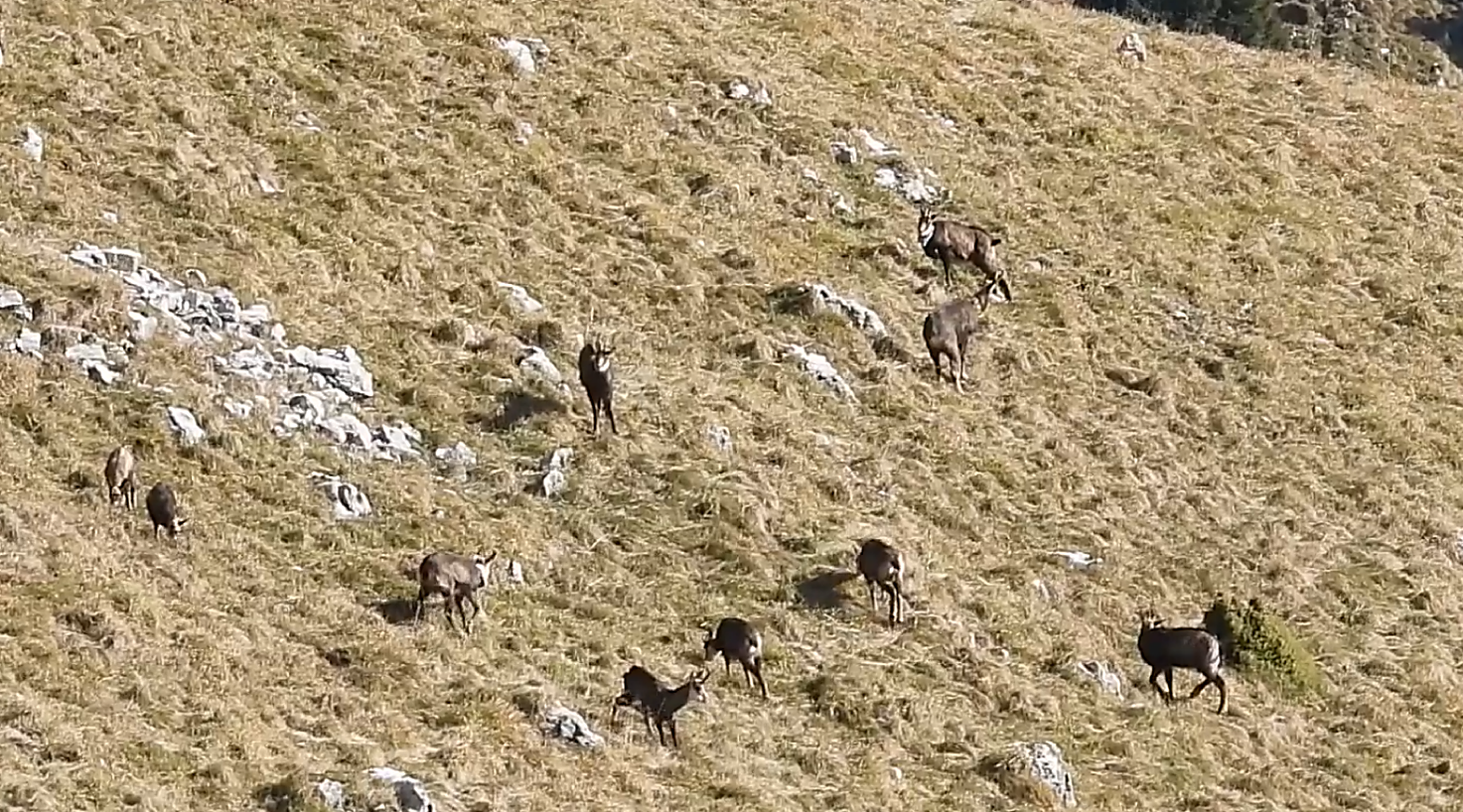

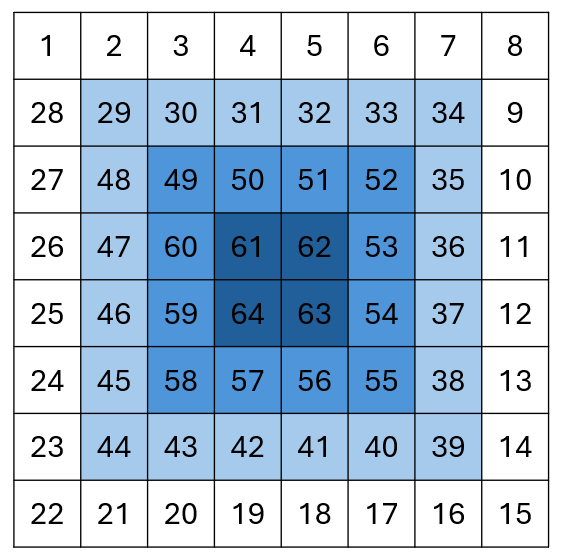


**Figure S3**. Example of an observed group of 10 chamois. The upper panel shows the original picture of the chamois group taken from a vantage point during an observation, while the lower panel shows the same picture with the virtual grid of Figure S3 virtually overlaid.
